# Photodynamic therapy for the treatment of primary cutaneous B-cell marginal zone lymphoma: A series of 4 patients

**DOI:** 10.1016/j.jdcr.2022.12.020

**Published:** 2023-01-25

**Authors:** Elise Toulemonde, Sarah Faiz, Romain Dubois, Marie Verhasselt-Crinquette, Olivier Carpentier, Henry Abi Rached, Laurent Mortier

**Affiliations:** a Department of Dermatology, Claude Huriez Hospital, Lille University Hospital, Lille, France; b Department of Dermatology, Hospital of Douai, Douai, France; c Department of Anatomopathology, Biology and Pathology Center Pierre-Marie Degand, CHU Lille, Lille, France; d Department of Dermatology, Hospital of Roubaix, Roubaix, France; e Department of Dermatology, Claude Huriez Hospital, CARADERM and University of Lille, U1189 Inserm, Lille, France

**Keywords:** cutaneous B-cell lymphoma, dermaroller, marginal zone lymphoma, methyl-aminolevulinate, microneedling, photodynamic therapy, primary Cutaneous B-cell lymphoma, CBCL, cutaneous B-cell lymphoma, MAL, methyl aminolevulinic, MZL, marginal zone lymphoma, PDT, photodynamic therapy, PpIX, protoporphyrin IX

## Introduction

Photodynamic therapy (PDT) aims to destroy targeted abnormal cells with the use of a photosensitizer that selectively accumulates in cancerous cells and metabolizes into protoporphyrin IX (PpIX) during incubation. The skin lesions are then exposed to a light source at a specific wavelength based on the absorption spectrum of PpIX.^1^ This results in a phototoxic reaction leading to the apoptosis of targeted cells.^2^

Two main photosensitizers are used: 5-aminlevulinic-acid and methyl-aminolevulinic (MAL). MAL is described as more lipophilic with deeper penetration of the skin and higher stability than 5-aminlevulinic-acid.^3^^,^^4^ MAL-PDT could possibly be more effective for the treatment of deep skin lesions.^3^

PDT is used in numerous non-melanoma skin cancers^5^^,^^6^ and in non-oncological diseases such as infectious or inflammatory pathologies.

A handful of studies have shown promising results of PDT in the treatment of early-stage mycosis fungoides.^7^^,^^8^ However, little data regarding PDT in cutaneous B-cell lymphomas (CBCL) have been published.^9^ We report a case series of 4 patients with marginal zone lymphoma (MZL)-type CBCL treated by PDT.

## Patients and methods

Four patients with MZL-type CBCL were treated with MAL-PDT. The patients’ characteristics are shown in Table I. The mean age was 51 years, ranging from 27 to 64. The sex ratio was 1 (2 females and 2 males). All the patients had multiple target skin lesions ranging from 5 to 7 thus PDT was preferred over surgical excision or radiotherapy. The diagnosis was established in all patients by routine histopathology and immunophenotyping on skin biopsy samples by a trained pathologist, with experience in cutaneous lymphomas. The patients were staged and confirmed to have skin limited diseases. Half of them had received at least 1 treatment prior to our study. The treatments were topical high potency corticosteroids (patient 2 and patient 3) and rituximab (patient 2 and patient 3). Both patients previously treated by rituximab had received 8 infusions prior to PDT.

**Table I:** Clinical characteristics of patients

Patient no	Age	Gender	Prior treatment lines	Number of skin lesions treated
1	64 y old	F	None	5
2	55 y old	M	Rituximab, clobetasol	7
3	61 y old	M	Rituximab, clobetasol	5
4	27 y old	F	None	7

We performed a micro-needle abrasion of the skin lesions by using a dermaroller to enhance drug penetration on the targeted skin lesions. We immediately applied a thin layer of MAL 168 mg/g 0.5 to 1 cm beyond the skin lesions.^10^ MAL was applied under light occlusive dressing for 2 and a half hours.

We then removed the dressing and the excess topical MAL and carried out illumination for 7 minutes and 30 seconds with the AKTILITE© device (Galderma) with a light dose of 37 J/cm^2^.^11^

Every patient received multiple sessions: patient 1 and patient 2 were treated initially every 2 weeks and patient 3 and patient 4 every 4 weeks. Treatment was carried out until 6 illuminations were completed. Early discontinuation was possible in case of a clinical response confirmed by histopathology.

## Results

**Fig 1:**
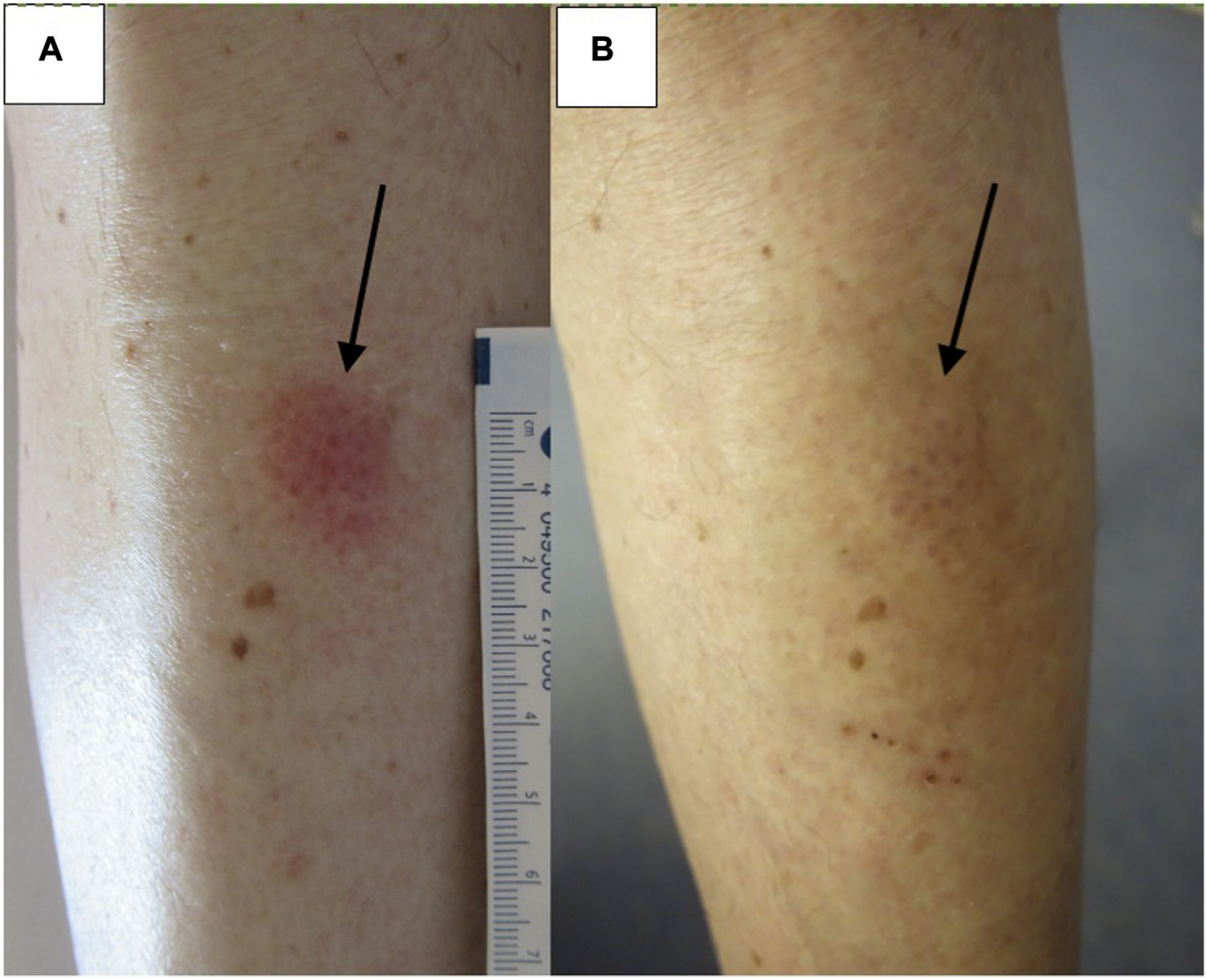
Cutaneous B-cell lymphoma of patient n° 1 (*arrows* pointing the skin lesion): (**A**) before second illumination. **B,** Before sixth illumination.

**Fig 2:**
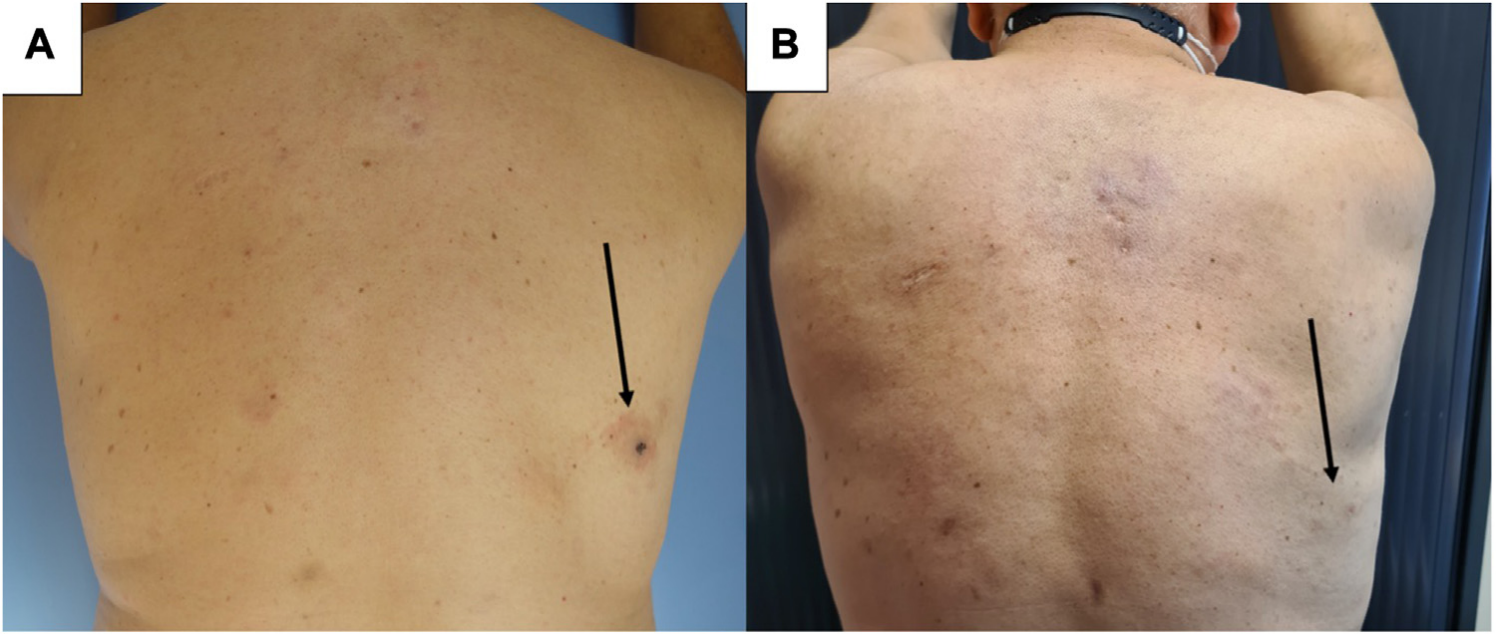
Cutaneous B-cell lymphoma of patient n° 2: (**A**) before the third illumination. **B,** Reevaluation 16 months after the last illumination (*arrows* pointing the skin lesion).

**Fig 3:**
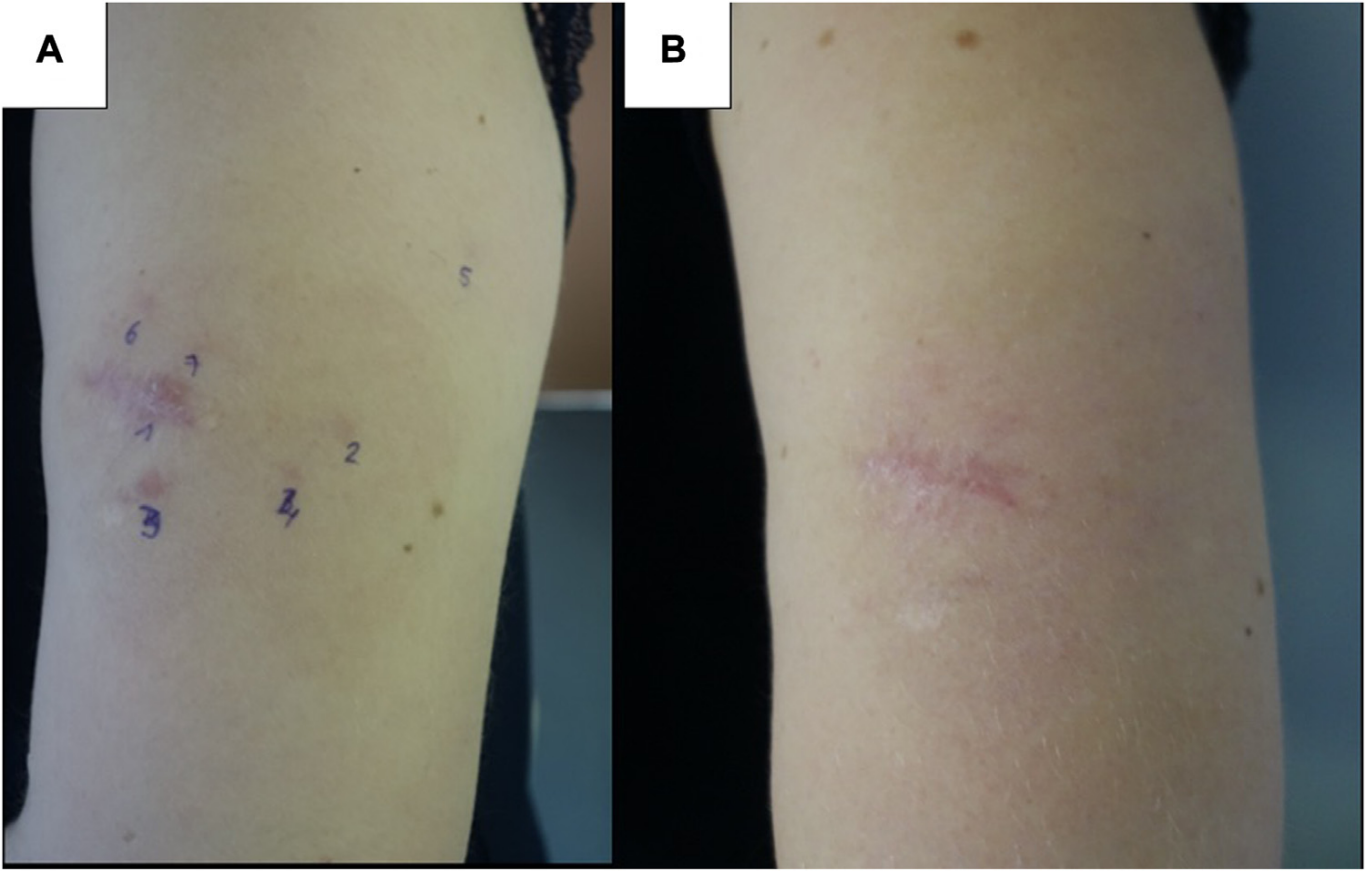
Cutaneous B-cell lymphoma of patient n° 4: (**A**) before second illumination. **B,** One month after fifth illumination.

**Fig 4:**
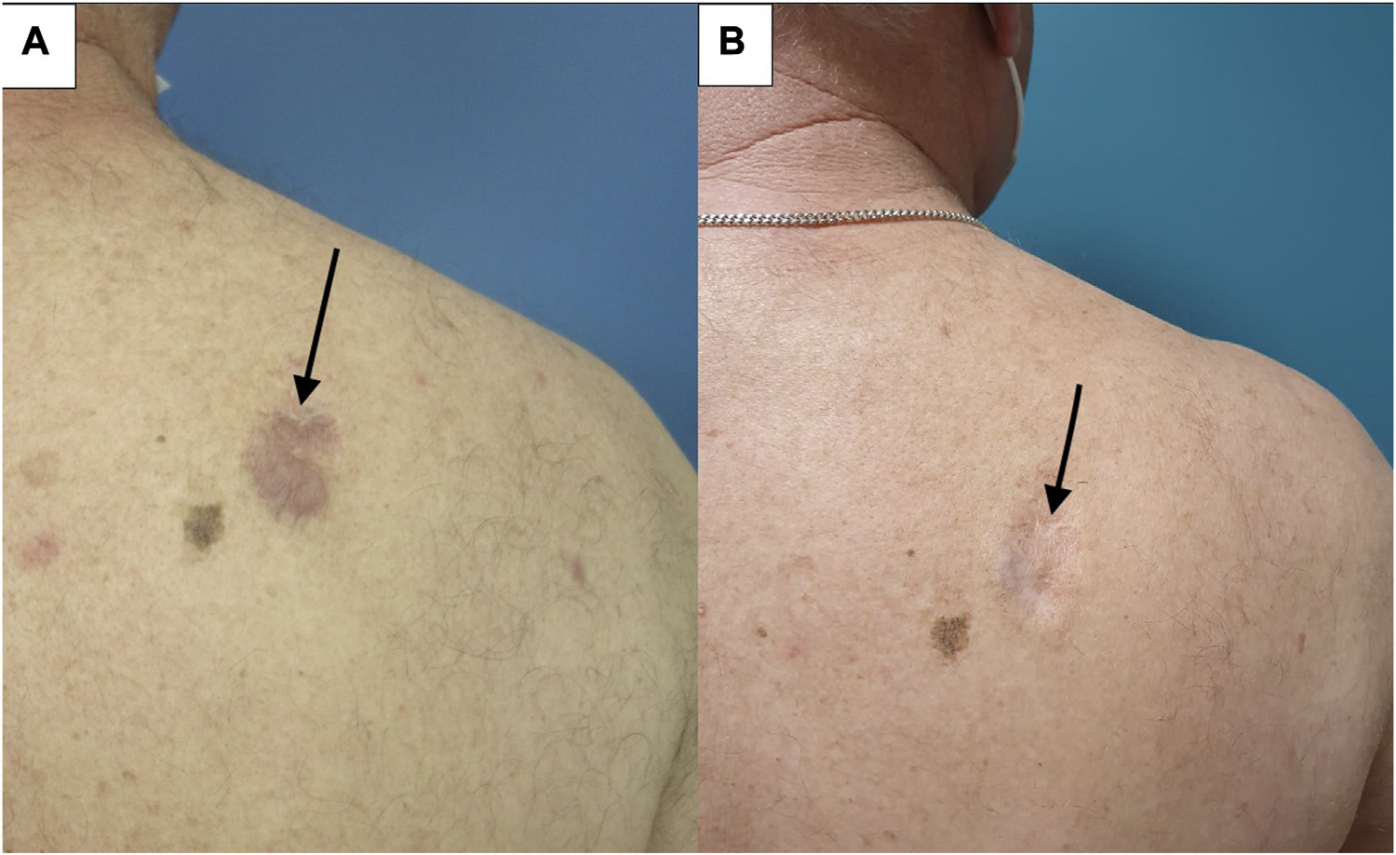
Cutaneous B-cell lymphoma of patient n° 3 (*arrows* pointing the skin lesion): (**A**) Before second illumination. **B,** during follow-up showing clinical response.

PDT showed effectiveness in all 4 patients of varying degrees, with both partial and complete responses in 1 or several lesions. Out of the 4 patients treated, 2 patients (50%) experienced clinical and histological remission of all treated skin lesions (patient 2 and patient 4) after 5 to 6 MAL-PDT sessions (Figs 2, *A* and *B*, and 3, *A* and *B*). Patient 3 showed clinical response, however a skin biopsy demonstrated histological evidence of persistent disease (Fig 4, *A* and *B*). Patient 1 had a mixed response, with complete resolution of 3 lesions (1 of which later relapsed) and stable disease in 2 others (Fig 1, *A* and *B*). During treatment and follow-up no new lesions were observed. The results are summarized in Table II.

**Table II:** Results to treatment

Patient no	Number of PDT sessions	Response to treatment	Duration of response
1	9	2 complete clinical response (Fig 1, *A* and *B*) 1 complete clinical response followed by tumor recurrence 2 partial clinical response	12 and a half months
2	5	Complete clinical and histological response	16 and a half months ongoing response
3	6	Complete clinical response with histological evidence of persistent disease (Fig 2, *A* and *B*)	16 months ongoing clinical response
4	5	Complete clinical and histological response (Fig 4, *A* and *B*)	3 and a half months

*PDT*, Photodynamic therapy.

The tolerance of treatment was moderate for each patient. Patient 1, however, had early termination during 2 sessions due to pain. Average pain scale was 4.15 ranging from 2 to 9 (based on a Visual Analog Scale score 0 to 10).

## Discussion

Several treatment options are available for CBCL, including radiotherapy, high potency topical corticosterois, local excision, intralesional steroids, topical imiquimod, and rituximab.^2^ Our study supports PDT as a potential treatment option for MZL-type CBCL.

Previous studies suggest effectiveness of PDT for the treatment of localized cutaneous T-cell lymphomas such as mycosis fungoides^7^^,^^8^ which led us to believe it could be a plausible treatment for MZL-type CBCL. The mechanism is based on an accumulation of PpIX in the lymphocytic infiltrate of CBCL after topical use of a photosensitizer^7^ and apoptosis of targeted cells during illumination.

A novel aspect of our treatment includes, a micro-needle abrasion of the skin lesions, with the use of a dermaroller, to improve efficacy by enhancing drug penetration as atypical lymphocytes are present in deep skin layers.^10^^,^^12^^,^^13^ Photosensitizers such as 5-aminlevulinic-acid poorly penetrate intact skin^13^ and therefore poorly metabolize to PpIX in the deeper dermis. Microneedling of the skin causes abrasion of the stratum corneum by creating micropores and increases penetration of topical drugs to the dermal layer.^3^^,^^12^^,^^14^ The use of microneedles prior to the application of photosensitizers has shown higher PpIX production.^15^

By increasing skin penetration of photosensitizers, it could boost the efficacy of PDT, lead to a reduction in dose and a decrease in potential side effects.^16^

To our knowledge, only 1 other study reports the efficacy of PDT in CBCL (1 follicle center, 2 MZL). Our 4 patients therefore add substantially to the current literature on this topic.^9^

We had close follow-up of our patients for a median of 14.25 months. Recurrence after treatment was seen in 1 patient: patient 4 had local recurrence of 3 lesions measuring 1 mm each, 3 and a half months after the last PDT illumination. PDT, similar to local treatments, like topical corticosteroids or imiquimod, treats visible lesions and does not prevent the risk of distant relapse. In some cases, efficacy of PDT can be seen after several months of treatment, as we observed in patient 3 who showed clinical response 4 months after the last PDT illumination. This highlights the need to pursue follow-up after treatment so as not to overlook delayed effectiveness of treatment as well as disease recurrence.

The limits of our study include the small number of patients, the variable illumination protocols based on the absence of codified protocols. Additionally, spontaneous remission of CBCL can be seen, therefore creating a possible confirmation bias. Moreover, 2 patients had a prior treatment by several cycles of rituximab which can take time to show effectiveness and could have altered presumed efficacy of PDT.

In our 4 cases, PDT was an effective treatment with mild and short-term side effects and could be considered a treatment option for patients with multiple lesions of MZL-type CBCL who fail topical therapy and prefer conservative treatment.

## Conclusion

PDT seems to be an interesting therapeutic option for localized MZL-type CBCL. Microneedles could increase the efficacy of treatment by enhancing drug penetration. However, illumination protocols are not codified. Further studies with larger series of patients are needed to validate this treatment and establish an optimal illumination scheme. Clinical examination must be pursued as recurrence of skin lesions have been seen.

## Conflicts of interest

None disclosed.
